# A common 56-kilobase deletion in a primate-specific segmental duplication creates a novel butyrophilin-like protein

**DOI:** 10.1186/1471-2156-14-61

**Published:** 2013-07-06

**Authors:** Johanna Aigner, Sergi Villatoro, Raquel Rabionet, Jaume Roquer, Jordi Jiménez-Conde, Eulàlia Martí, Xavier Estivill

**Affiliations:** 1 Bioinformatics and Genomics Program, Centre for Genomic Regulation (CRG), Barcelona 08003, Spain; 2 Universitat Pompeu Fabra (UPF), Barcelona 08003, Spain; 3 Centro de Investigación Biomédica en Red en Epidemiología y Salud Pública (CIBERESP), Barcelona 08003, Spain; 4 Hospital del Mar Medical Research Institute (IMIM), Barcelona 08003, Spain; 5 Neurology Department, Neuvovascular Research Group, Institut Hospital del Mar d'Investigacions Mèdiques (IMIM), Barcelona 08003, Spain; 6 Universitat Autònoma de Barcelona (UAB), Barcelona 08003, Spain

## Abstract

**Background:**

The Butyrophilin-like (BTNL) proteins are likely to play an important role in inflammation and immune response. Like the B7 protein family, many human and murine BTNL members have been shown to control T lymphocytes response, and polymorphisms in human *BTNL2* have been linked to several inflammatory diseases, such as pulmonary sarcoidosis, inflammatory bowel disease and neonatal lupus.

**Results:**

In this study we provide a comprehensive population, genomic and transcriptomic analysis of a 56-kb deletion copy number variant (CNV), located within two segmental duplications of two genes belonging to the BTNL family, namely *BTNL8* and *BTNL3.* We confirm the presence of a novel BTNL8*3 fusion-protein product, and show an influence of the deletion variant on the expression level of several genes involved in immune function, including *BTNL9,* another member of the same family. Moreover, by genotyping HapMap and human diversity panel (HGDP) samples, we demonstrate a clear difference in the stratification of the *BTNL8_BTNL3-del* allele frequency between major continental human populations.

**Conclusion:**

Despite tremendous progress in the field of structural variation, rather few CNVs have been functionally characterized so far. Here, we show clear functional consequences of a new deletion CNV (*BTNL8_BTNL3-del)* with potentially important implication in the human immune system and in inflammatory and proliferative disorders. In addition, the marked population differences found of *BTNL8_BTNL3-del* frequencies suggest that this deletion CNV might have evolved under positive selection due to environmental conditions in some populations, with potential phenotypic consequences.

## Background

The human genome has been shown to be quite plastic, with many regions presenting gains and losses of genetic material amongst individuals, also known as copy number variants (CNVs) [1]. Both germline and somatic CNVs have been found to play important roles in several disorders, including neuropsychiatric, infectious and autoimmune diseases, and cancer [2-5]. In addition, variation in copy number has been shown to be a major driving force in evolution, especially within the primate lineage. Compared to other mammals, such as rat and mouse, the genome of humans and great ape species is characterized by a significant enrichment in CNVs [6]. A high percentage of these variants (25%-50%) are located in close proximity to or are part of segmental duplications (SDs), also called low-copy repeat (LCRs) elements, which present blocks of highly (>95%) identical sequences generated during primate evolution [7,8]. Regions enriched in LCRs predispose to genomic rearrangement during meiosis, originated by non-allelic homologous recombination (NAHR) between repetitive LCR elements [9]. Interestingly, CNVs that overlap with SDs have been shown to be especially rich in gene and pseudogene content, and therefore are likely to be of clinical importance. Moreover, genes with functions related to immunity and infection are enriched in CNVs, and most primate-specific hotspots for CNVs formation have been found in genes with roles in immune or environmental response, suggesting an association between CNVs and SDs in human health [10,11].

The precise structure of many CNVs and their potential functional consequences are still largely unknown. In the present study, by using lymphoblastoid cell lines (LCL) derived from human subjects of European ancestry, we undertook a systematic genetic, gene expression and evolutionary analysis of a previously uncharacterized 56-kb deletion CNV, located on the subtelomeric region of human chromosome 5q35.3, in a cluster with several genes encoding tripartite motif-containing (TRIM) proteins and genes involved in the olfactory system. We show that the breakpoints of the deletion are located within two primate-specific SDs of two genes belonging to the butyrophilin-like (BTNL) protein family, and that the polymorphic deletion (*BTNL8_BTNL3-del* allele) leads to the formation of a new fusion gene (BTNL8*3). We confirm the presence of a novel BTNL8*3 fusion-protein product, and show an influence of the deletion variant on the expression level of neighbouring gene *BTNL9* and several other genes involved in immune response and cancer, thus suggesting an involvement of this CNV in specific biological pathways. Moreover, we have found differences in the frequency of the deletion allele amongst major continental ethnic groups, being rare in African and Oceanic populations but common in Asians, Americans and Europeans. After carefully looking for tagging single nucleotide polymorphism (SNPs) that could be used as a surrogate for the deletion, we only were able to identify a suitable tag SNP in some populations tested, suggesting that the CNV is a recent and recurrent event in humans. Taken together, our findings show functional consequences of a novel deletion polymorphism with impact in world population’s distribution and potential implications in physiological processes of immune response and proliferation.

## Results

### Array-CGH analysis of chromosome 5q35.3 reveals a 56-kb polymorphic deletion

**Figure 1:**
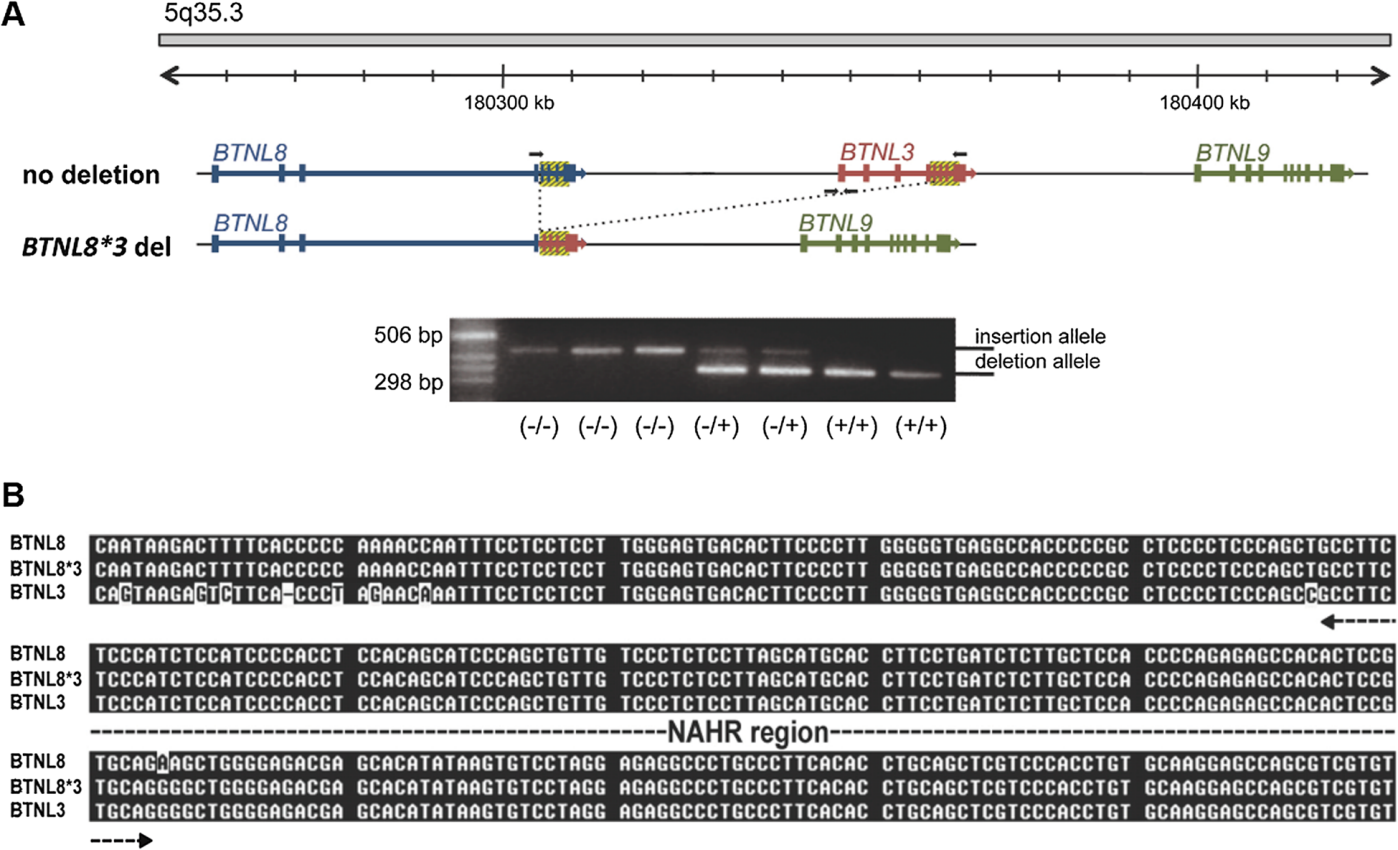
**Region of the BTNL gene cluster containing the BTNL8_BTNL3-del allele and sequence structure of deletion. (A)** The figure shows 200-kb of the genomic region on human chromosome 5. The expected transcripts of the three genes in this region are shown with their transcript orientation. The CNV region, which is indicated by the dashed line, removes 56-kb, from intron four of BTNL8 to intron four of BTNL3. The black-yellow dashed box represents the SD shared between BTNL8 and BTNL3. PCR assays were designed to distinguish between non-deleted and deleted alleles (short arrows show position of PCR oligonucleotides). The PCR assay amplifies a ~420-bp product for the non-deleted allele, and a ~340-bp fragment for the deleted allele (bottom). Results from the PCR assay are shown for seven LCLs. **(B)** The deleted allele shows long homologous stretches at its breakpoints (~1.6-kb sequence blocks of 98% identity, of which 300-bp are shown aligned here) and thus probably results from non-allelic homologous recombination (NAHR). The “cross-over” occurred somewhere within 112-bp of identical sequence indicated as “NAHR-region”.

By using the human Agilent 244 K whole-genome array and consequent customized Multiplex Ligation-dependent Probe Amplification (MLPA) analysis of LCLs derived from HapMap samples, we found a potential CNV, located on chromosome 5q35.3. We next developed a direct PCR amplification assay to map the breakpoints of the CNV, finding that it consists of a ~56-kb deletion polymorphism (chr5:180375027–180430596 in hg19) recently reported by Kim et al. [12]. The CNV removes the genomic sequence between intron four of *BTNL8* and intron four of *BTNL3*, resulting in a new BTNL8*3 fusion gene without any alterations in the coding sequence (Figure 1A). The breakpoints of the deleted allele are located in two ~1.6-kb SD that share 98% identity and are separated by ~55,570 nucleotides on the *BTNL8* and *BTNL3* genes, indicating that the deletion resulted from a NAHR event. Each 1.6-kb SD contains parts of intron four to exon eight of *BTNL8* and *BTNL3.* The crossover occurred somewhere within 112-bp of identical sequence of these two genes in intron four (Figure 1B).

### Linkage disequilibrium with SNPs in the genomic region

To evaluate linkage disequilibrium (LD) between *BTNL8_BTNL3-del* and tag single nucleotide polymorphisms (SNPs) covering the *BTNL8* and *BTNL3,* surrounding haplotype structure, we genotyped the 56-kb deletion in 1,103 unrelated individuals included in the HapMap Phase III project and searched for potential tagging SNPs, based on the genotypes on the HapMap data release 28 (http://hapmap.ncbi.nlm.nih.gov), and an independent control cohort of 2,000 Spanish unrelated individuals. Several SNPs were in high LD (r^2^ > 0.9) with the deletion in the CEU, CHD, CHB, MEX, GHI and JPT sets. The best taggers in these populations were rs2387715 and rs4700772 (CEU, CHB, JPT, r^2^ = 1; MEX, r^2^ = 0.95; CHD, r^2^ = 0.96; and GHI, r^2^ = 0.94). Finally, in the Tuscan population only one SNP was selected as a tagger, rs10063135 (TSI, r^2^ = 0.81). In contrast, we could not identify a proxy SNP (r^2^ > 0.8) in African-ancestry populations (ASW, LWK, MKK or YRI). In fact, the SNPs in higher LD in these populations were: rs3733755 (YRI, r^2^ = 0.38); rs2387715 (ASW, r^2^ = 0.69; MKK, r^2^ = 0.32); and rs4701016 (LWK, r^2^ = 0.36) (Table 1). Based on these LD results, we selected three of these SNPs (rs3733755, rs10063135 and rs4700772) for genotyping 2,000 Spanish samples, and none of them showed strong LD (r^2^ > 0.8) with the deletion in our dataset: rs10063135, r^2^ = 0.63; rs3733755, r^2^ = 0.63, and rs4700772 r^2^ = 0.724.

**Table 1:** List of SNPs tested for LD with the BTNL8*3 deletion CNV

			**ASW**		**CEU**		**CHB**		**CHD**		**GHI**		**JPT**		**LWK**		**MKK**		**MEX**		**TSI**		**YRI**	
**rs Number**	**Position**	**Distance to deletion breakpoints**	**D'**	**r**^**2**^	**D'**	**r**^**2**^	**D'**	**r**^**2**^	**D'**	**r**^**2**^	**D'**	**r**^**2**^	**D'**	**r**^**2**^	**D'**	**r**^**2**^	**D'**	**r**^**2**^	**D'**	**r**^**2**^	**D'**	**r**^**2**^	**D'**	**r**^**2**^
rs1904435	180322735	52,292					0.94	**0.834**	1	0.77	0.81	0.596	1	**0.829**	0.31	0.03			0.93	0.537	0.8	0.495	0.69	0.099
rs7735361	180329359	45,668	1	0.086	0.91	**0.843**	1	0.658	1	0.706	0.94	0.663	1	**0.796**	0.3	0.034	0.37	0	0.82	0.58	0.8	0.54	0.03	0
rs17704291	180336849	38,178	0.8	0.43	1	**0.96**	0.94	**0.889**	-	-	-	-	1	**1**	0.28	0.04			0.94	0.76	0.9	0.68	0.3	0.06
rs4700772	180341845	33,182	0.8	0.539	1	**1**	1	**1**	1	**0.966**	1	**0.946**	1	**1**	0.8	0.25	0.63	0.322	1	**0.952**	1	0.752	0.8	0.3
rs2387715	180361266	13,761	0.8	0.694	1	**1**	1	**1**	1	**0.966**	1	**0.944**	1	**1**	0.8	0.237	0.63	0.322	1	**0.952**	1	0.749	0.8	0.33
rs3733755	180374484	543	1	0.686	1	**0.815**	1	0.692	1	0.451	1	0.588	1	0.486	1	0.31	0.77	0.195	1	**0.86**	1	0.598	1	0.38
deletion	180375027-180430596																							
rs10063135	180432024	1,428	1	0.177	1	**0.923**	1	**0.946**	1	**0.966**	1	0.545	1	0.754	0.4	0.161	0.8	0.101	1	**0.82**	0.97	**0.81**	0.8	0.09
rs4700774	180434216	3,620	1	0.473	1	**1**	1	**0.945**	1	**0.966**	1	**0.893**	1	1	0.84	0.12	0.5	0.09	0.95	**0.903**	1	0.752	0.2	0.07
rs7721042	180439488	8,892			1	**0.958**	1	**0.939**	-	-	-	-	1	1	-	-					-	-	0.03	0
rs11249756	180455372	24,776	1	0.473	1	**0.923**	1	**0.945**	0.96	**0.86**	0.8	0.64	1	1	0.4	0.161	0.44	0.07	0.94	**0.807**	0.94	0.72	0.2	0.07
rs4701016	180458539	27,943	1	0.686	0.91	0.733	1	0.646	0.86	0.37	0.6	0.3	1	0.486	1	0.364	0.2	0.01	0.85	0.719	0.9	0.55	1	0.3
rs6868418	180458813	28,217	1	0.686	0.91	0.733	1	0.646	0.86	0.4	0.6	0.3	1	0.486	1	0.364	-	-	-	-	-	-	1	0.3

HapMap phase III populations were screened for tagging SNPs for the *BTNL8_BTNL3-del* allele. Several SNPs could be identified in Eastern Asian (CHB, CHD and JPT), American (GIH and MEX) and CEU population. However, no LD could be observed in African population (ASW, LKW, MKK and YRI) and only a weak association for rs10063135 could be detected in TSI population.

r^2^ higher than0.8 indicates a significant correlation. ASW, individuals with African ancestry from Southwest USA; CEU, individuals from Utah with northern and western European ancestry; CHB, Han Chinese from Bejing; CHD, Han Chinese from Denver, Colorado; GIH, Gujarati Indians from Houston, Texas; JPT, Japanese from Tokyo; LWK, Luhya from Webuya, Kenya; MEK, individuals with Mexican ancestry from Los Angeles, California; MKK, Maasai from Kinyawa, Kenya; TSI, Toscans from Italy; YRI, Yorubas from Ibadan, Nigeria.

### Population differences in the frequency of the *BTNL8_BTNL3-del* allele

To check whether population-based differences exist for the *BTNL8_BTNL3-del* allele, HapMap samples from 120 CEU; 90 CHB; 90 JPT; 120 YRI; 43 ASW; 100 CHD; 100 GIH; 100 LWK; 90 MEX; 150 MKK; 100 TSI; and 2,000 Spanish samples were genotyped. In addition, we analyzed 1,007 samples from 39 ethnical groups of the Centre d’Etude du Polymorphisme Humain (CEPH) Human Genome Diversity Panel (HGDP) (Additional file 1: Table S1 and Table S2).

**Figure 2:**
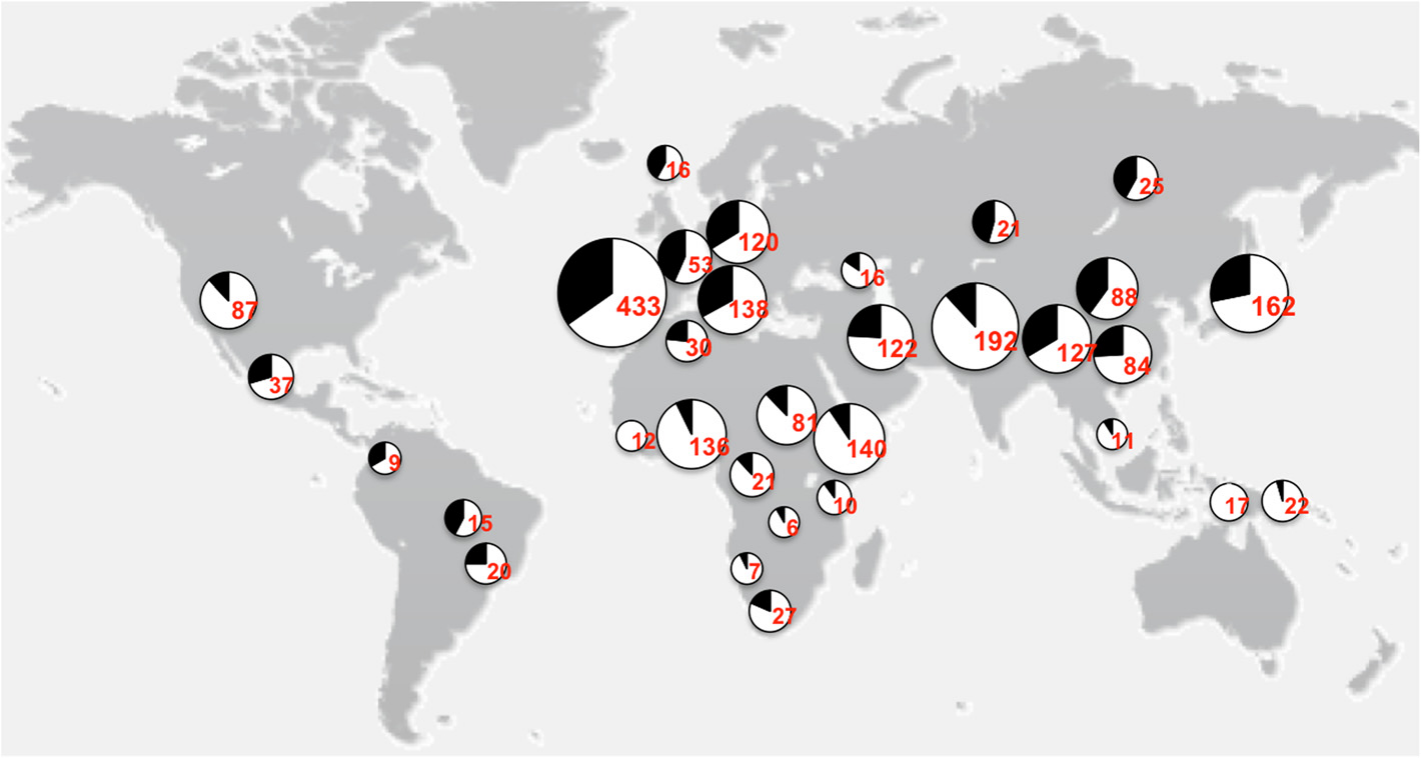
**World-wide distribution of the *BTNL8_BTNL3-del *allele in different human populations.** Genotype analysis of populations is shown over a map of the world. The deletion frequency is indicated by the black part of the pie in the chart. Total number of individuals genotyped is given in red.

We observed clear differences in allele frequencies between major continental groups. The deletion is significantly underrepresented in Oceanic, central Asian and Sub-Saharan African populations but significantly overrepresented in European and American populations. In CEU population, 12% are homozygous for the deletion allele and 41% carry at least one *BTNL8_BTNL3-del* allele, corresponding to an allele frequency of 34%. In the CHB-JPT population the deleted allele shows a frequency of 29%, while in African and Oceanic populations show a significantly reduced frequency for the *BTNL8_BTNL3-del* allele of 9% and 3%, respectively (Figure 2, Additional file 1: Table S3). In order to rule out large-scale genotyping errors, we calculated estimates of Hardy-Weinberg equilibrium for each of the 39 HGDP populations, as well as for all continental HapMap and HGDP groups, but could not observe any significant deviations except for Hezhen (China), Cambodian and Orcadian population, which most likely is due to the small sample size (10, 10 and 15 individuals, respectively) sequenced (Additional file 1: Table S3 and Table S4).

### The BTNL8*3 56-kb deletion leads to a new chimeric transcript and protein product

**Figure 3:**
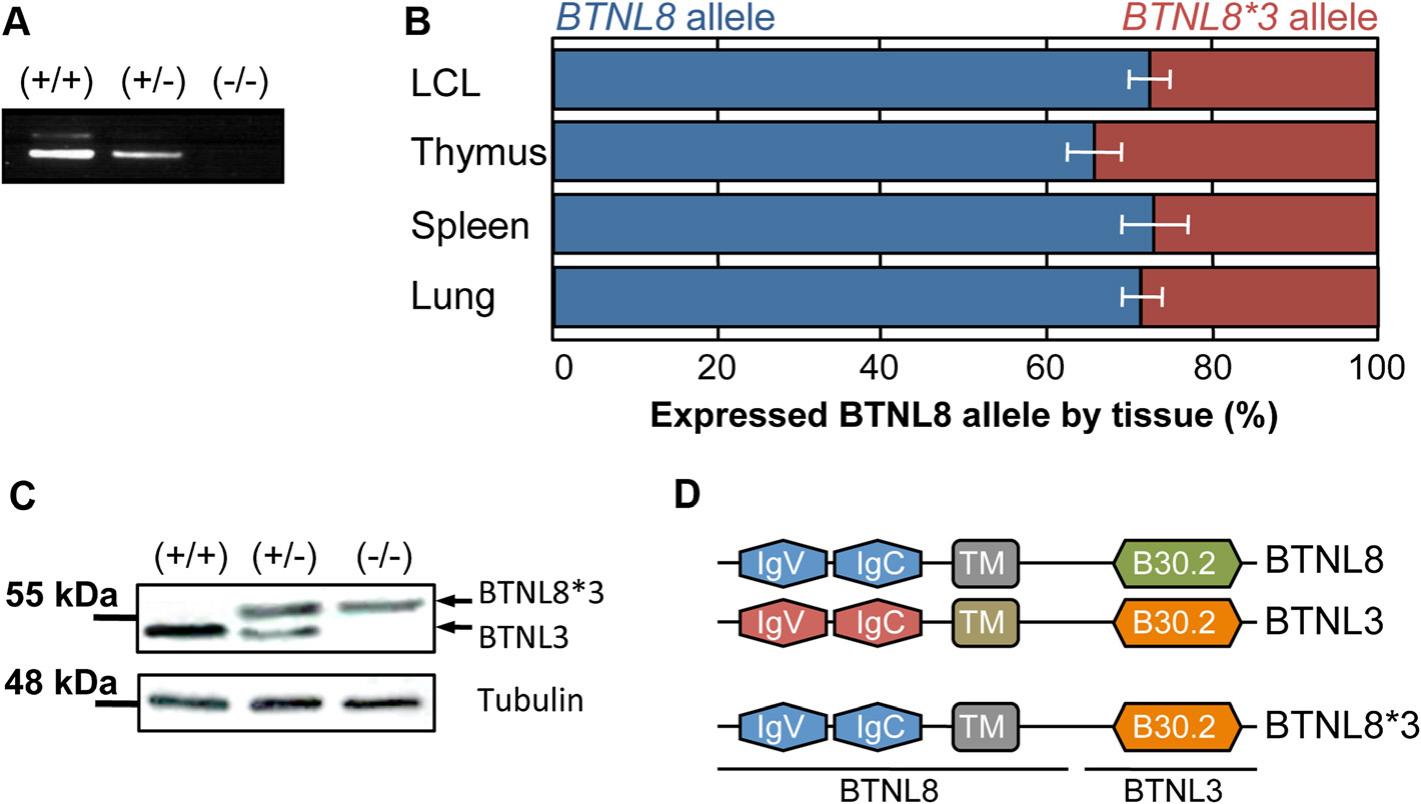
Identification of a chimeric BTNL8*3 product. (A) In LCLs carrying one or two copies of the *BTNL8_BTNL3-del* allele, a fusion BTNL8*3 mRNA product could be detected by RT-PCR. **(B)** Allelic expression differences are shown in individual tissues. In tissues heterozygous for the BTNL8*3 allele, the non-deleted *BTNL8* allele was expressed threefold higher than the BTNL8*3 allele (all tissues combined). **(C)** Representative western blot analysis revealed a novel protein in LCL cells expressing the deleted allele. The band intensities were normalized to tubulin expression. **(D)** The putative protein resulting from the BTNL8*3 deletion CNV would contain the extracellular IgV-like and IgC-like domains and the TM domain of BTNL8 and the cytoplasmic B30.2 domain of BTNL3.

To further analyse the CNV at the transcriptional level, LCLs were genotyped and RT-PCR analysis and subsequent sequencing was carried out on samples containing none, one or two copies of the *BTNL8_BTNL3-del* allele. In cell lines heterozygous and homozygous for the deletion CNV we detected a new, in-frame, fusion transcript, BTNL8*3, which consisted of the juxtaposition of exons one to four of *BTNL8* to exons five to eight of *BTNL3* (Figure 3A). Thus, the predicted BTNL8*3 transcript would contain the 3′-UTR from *BTNL3,* but the *BTNL8* upstream regulatory signals. In a next step, allele-specific quantitative RT-PCR (qPCR) analysis was performed on LCL and tissue samples heterozygous for the *BTNL8_BTNL3-del* allele. A chimeric BTNL8*3 transcript could be identified in all samples (Figure 3B). However, the fusion transcript was present at a significantly reduced level, compared to *BTNL8* mRNA with which it shares its complete upstream regulatory region. Moreover, western-blot analysis revealed the presence of a novel BTNL8*3 fusion protein in LCLs carrying the deleted allele, indicating the presence of a novel protein (Figure 3C). BTNL8 and BTNL3 share 68.5% similarity in their protein sequences (Additional file 1: Figure S1). The resulting chimeric protein would contain the extracellular Ig-like C and Ig-like V domain, and the transmembrane domain of BTNL8 and the intracellular B30.2 domain at its C-terminus (Figure 3D).

### BTNL8*3 CNV affects expression of neighboring gene *BTNL9*

**Figure 4:**
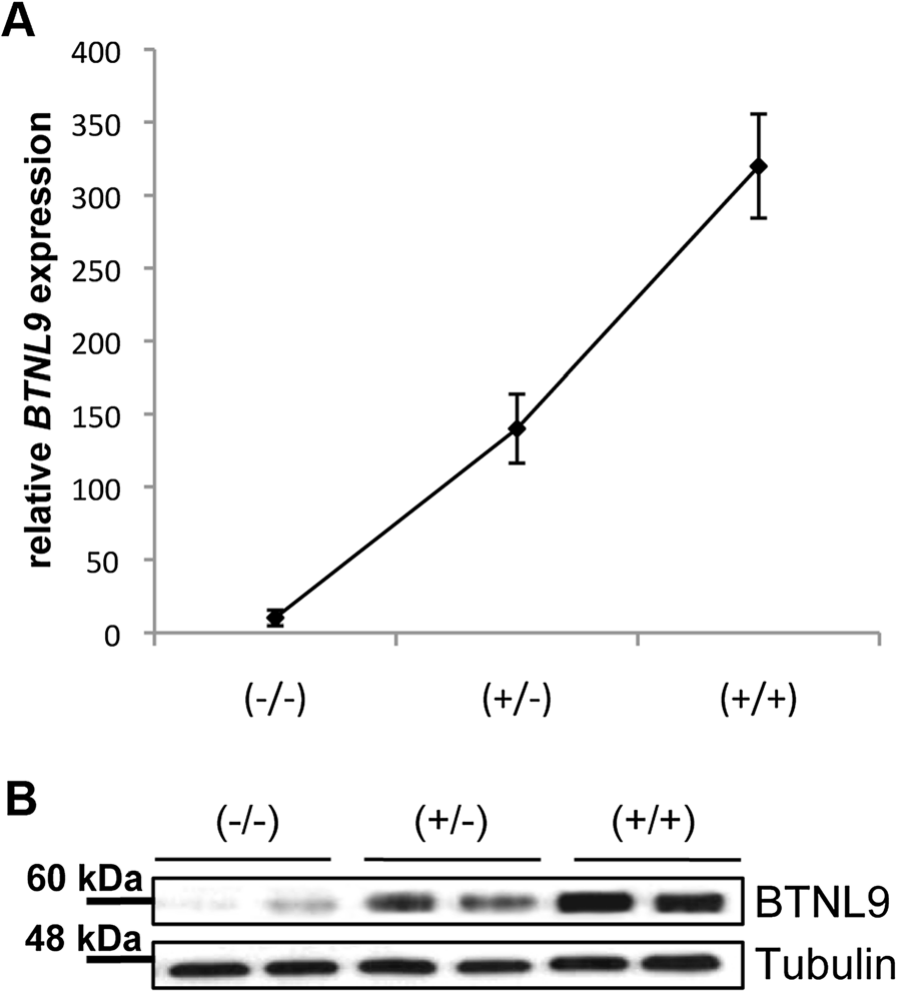
**Effect of *BTNL8-BTNL3_del *on BTNL9 level in LCLs. BTNL9 mRNA and protein levels were measured by RT-qPCR and western-blot analysis, respectively. (A)** For each genotype expression-level of five different samples were measured. Samples containing at least one deleted allele show significant reduced amount in *BTNL9* mRNA level. **(B)** Representative western-blot shows expression of BTNL9 in cell homozygous for the deletion versus cells homozygous for the non-deleted allele. The band intensities were normalized to tubulin expression.

*BTNL8* and *BTNL3* form a cluster together with another gene of the same family, *BTNL9*. Both, the *BTNL9* gene and its promoter region are intact in the *BTNL8_BTNL3-del* allele, thus *BTNL9* expression would not be expected to be affected by the BTNL8*3 deletion CNV. However, it is known that genomic neighborhoods may influence the expression level of a gene by a positional effect. This can be achieved by affecting *cis-*regulatory elements, such as transcription factor binding sites, or by re-organization of chromosomes into territories within the nucleus [13,14]. To test whether this is the case for *BTNL9* and other genes located behind the deletion CNV, we measured *BTNL9*, *TRIM7* and *TRIM41* mRNA expression level in 30 LCLs (10 of each genotype) by qPCR analysis. Expression of *BTNL9* could be detected at a moderate level in all cell-lines homozygous for the wild-type allele. In contrast, cell-lines heterozygous for the deletion expressed *BTNL9* at a significantly decreased amount, and in cell-lines homozygous for the deletion *BTNL9* expression was almost not detectable (Figure 4A). In line, western-blot analysis revealed a strong decrease in protein expression levels in cell lines heterozygous or homozygous for the *BTNL8_BTNL3-del* allele (Figure 4B). In addition, a slight but not significant effect could be observed at the expression-level of TRIM7, which is located ~200-kb from the deletion (data not shown), implying that indeed it is due to a positional effect, although other possibilities cannot be ruled out yet.

### The BTNL8*3 deletion CNV affects expression of several genes involved in immune response, cancer and developmental disorders

**Figure 5:**
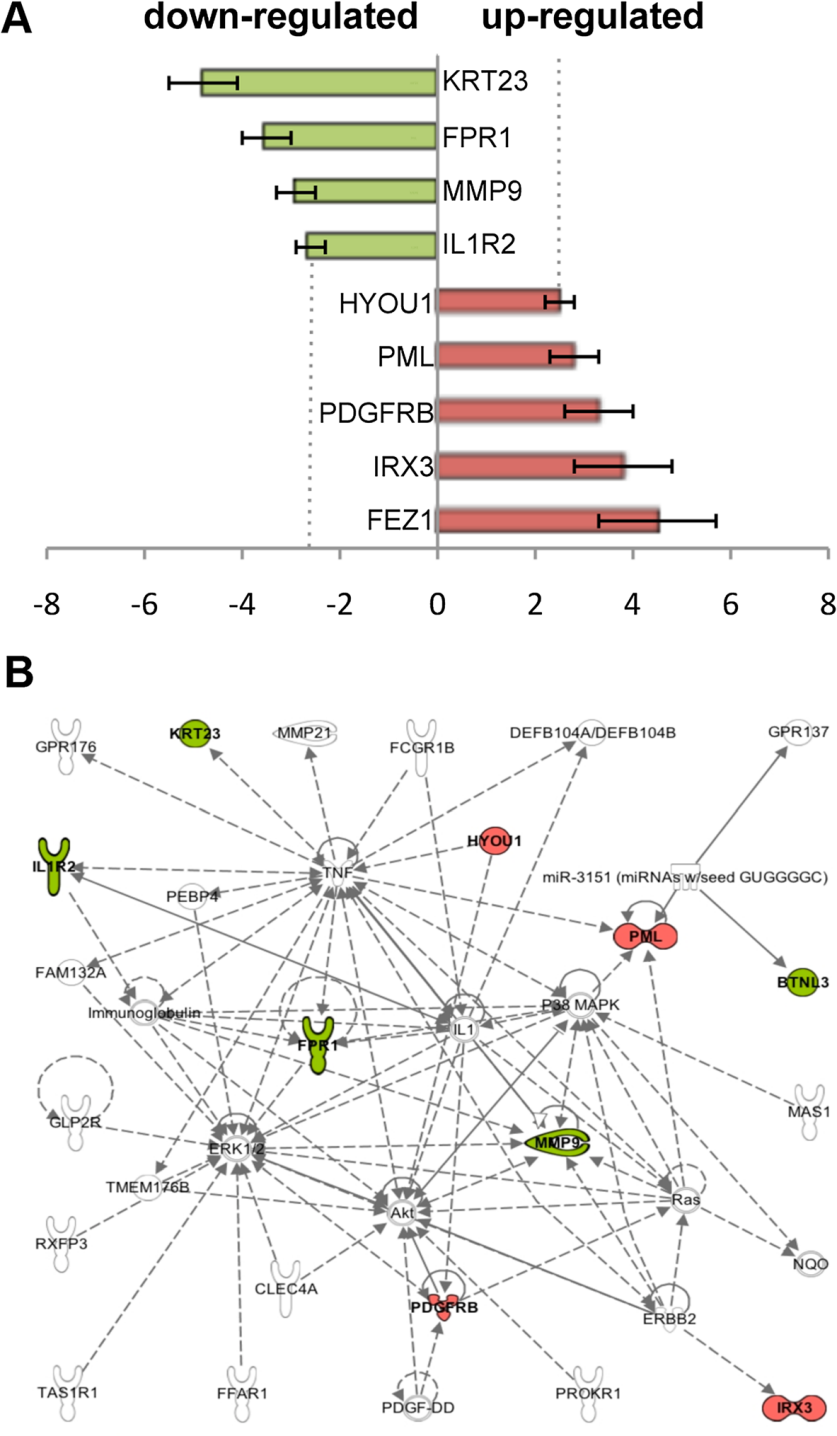
**Differential expression of genes depending on BTNL8/BTNL3 genotype. (A)** Nine genes could be confirmed by RT-qPCR analysis to be differentially expressed in LCLs homozygous for the *BTNL8_BTNL3-del* allele. Red, increased mRNA level; green, decreased mRNA level in LCLs homozygous for the CNV. **(B)** Network of genes formed by Ingenuity Pathway Analysis. Genes depicted in red (increased mRNA level) and green (decreased mRNA level) were confirmed by RT-qPCR analysis to be differentially expressed in LCLs homozygous for the CNV. Genes depicted in white were not found to be deregulated even though they are part of the same network.

Next we looked at potential downstream targets by utilizing transcriptional data from the Illumina genome-wide expression arrays of LCLs from 56 unrelated individuals, derived from CEU population [15]. Of the 56 cell-lines tested, 25 were homozygous for the insertion allele, 18 were heterozygous and 13 were homozygous for the deletion allele. In total, we found 20 genes showing a significant (p < 0.05) differential expression according to the *BTNL8/BTNL3* genotype (data not shown). Unfortunately, none of the genes deregulated by the CNV identified earlier, *BTNL9* and *TRIM7*, were expressed at a detectable level. To verify the microarray data, we performed qPCR analysis on 10 LCLs homozygous for the wild-type allele and 10 LCLs homozygous for the deletion allele and tested all 20 genes found deregulated at the microarray. Of the 20 genes, nine could be validated as differentially expressed in LCLs homozygous for the *BTNL8_BTNL3-del* allele (Figure 5A).

In a next step, we submitted the data to Ingenuity Pathway Analysis (IPA) for functional classification and to check whether these genes interact with each other in regulatory networks and biological pathways. In addition we included the genes directly affected by the CNV; *BTNL8*, *BTNL3* and *BTNL9*. The top functions mapped by IPA out of the 13 genes were: Hematopoiesis (3 genes, p < 4.27E-05), Immune Cell Trafficking (4 genes, p < 4.27-05), Hematological System Development and Function (5 genes, p < 7.93E-05), Tissue Morphology (7 genes, p < 2.40E-04) Connective Tissue Development and Function (3 genes, p < 3.08E-04). In addition, IPA formed one well-defined network, with function in cell morphology, cellular development and embryonic development, with nine mapped genes involved (Figure 5B). It should be noted that so far little is known about the function and operation mechanism of BTNL8, BTNL3 and BTNL9. Therefore they are not sufficiently well annotated for pathway analysis by Ingenuity Pathway.

## Discussion

CNVs are thought to significantly contribute to population-based adaptive evolution due to variability in expression levels. [16]. Especially recently emerged genes may have significantly contributed to evolutionary change and phenotypic adaptation in more recently diverged evolutionary lineages [17]. However, despite intensive work in the field, most CNVs and new resulting genes are so far, overall, rather poorly characterized at the functional level.

In this study, we provide a detailed functional analysis of a novel 56-kb deletion CNV involving two primate-specific genes of the *BTNL* family, namely *BTNL8* and *BTNL3*, who share 80% homology in their coding sequence. The CNV has arisen in two 1.6-kb SD blocks of 98% identity, suggesting that the deletion occurred due to a NAHR event. CNVs arisen from NAHR tend to recur due to the unstable nature of the large and highly identical SD, leading to variants of different haplotypes that share common endpoints. Multiple genomic disorders as Williams-Beuren syndrome, Charcot-Marie Tooth disease, schizophrenia, and developmental delay have been assigned to NAHR events between misaligned LCRs [18-21], making NAHR the most common mechanism mediating recurrent microdeletions and microduplications.

By carefully looking at SNPs data of the International HapMap project and through our own genotyping data, we found currently existing frequency-matched SNPs in LD (r^2^ > 0.8) with the BTNL8*3 CNV in Eastern Asian, American and northern European populations. In contrast, no LD could be detected in African, Italian and Spanish populations. Especially African populations have been shown to be highly heterogeneous and to significantly vary in their haplotype structure and LD from other populations [22]. However, also southern European subgroups as Italian, Spanish and Greek, have been shown to differ in their haplotype composition from northern and western European populations [23]. This most likely results from large and constant migratory influences and admixture with other European and North African populations. These substantial differences between northern and southern European subgroups imply that many variations found through genome-wide association studies (GWAS) studies carried out on northern and western European population may not be replicated in southern European subgroups. Moreover, the absence of a tagging SNP in some populations has important implications for the interpretation of association studies. It stresses that unless a direct genotyping assay is applied for the BTNL8*3 CNV, its potential phenotypic impact may be overlooked in GWAS.

The *BTNL8_BTNL3-del* allele has been found at higher frequencies in European, East Asian and American populations, as compared to African, Oceanic and Middle Eastern populations. Ethnicity plays an important role in inter-individual variability of the immune system. Through recurrent exposure to different pathogens, ethnic groups have selected genetic adaptations that provide resistance or reduced susceptibility to infection [24] meaning that many times CNVs result in an advantageous phenotype for some populations [25,26]. An example of a reduction in copy number being beneficial has been suggested for the α-globin locus, where it increases resistance to malaria infection and susceptibility to mild α-thalassemia. In regions where malaria is endemic, the deleted form of the α-globin gene can be found at an unusually high degree [27,28]. Other examples where the number of gene copies positively correlates with infection are *FCGR3B* and *DEFB4* genes, which are associated with glomerulonephritis, and Crohn’s disease, respectively [29,30]

Here we show that, in accordance with the partial deletion of these genes, the BTNL8 and BTNL3 proteins were not expressed in LCLs homozygous for the deletion, and they were found at a significantly reduced amount in LCLs carrying one deleted allele. RT-PCR analysis, sequencing and western blot analysis revealed a new BTNL8*3 fusion product without any alterations in the coding frame, suggesting the existence of a novel, functional protein. In addition, BTNL9, another member of the butyrophilin family, was down-regulated in LCLs carrying the *BTNL8_BTNL3-del* allele. However, the function of BTNL8, BTNL3 and BTNL9 is yet unknown; therefore, here we can only speculate what might be the consequence of the deletion.

The BTNL proteins belong to the butyrophilin (BTN) family. The eponymous BTN protein (BTN1A1) is a type I transmembrane glycoprotein whose expression is restricted to the mammary gland during lactation, where it is involved in the secretion of milk fat globules [31]. All BTN family members contain a signal peptide at their N-terminus, two Ig-like-domains (IgV and IgC) and a transmembrane-domain. The extracellular domain shows structural similarities to those of the B7 family, a protein family of co-stimulatory molecules involved in T cell activation [32]. In contrast to B7 proteins, most BTN members harbour a cytoplasmic B30.2 domain at their C-terminus. Like the B7 protein family, several human and murine BTN and BTNL family members have been shown to control T cell response [33]. While the B7 family of ligands and their receptors can regulate T cell response either through their positive (e.g. B7-1, B7-2, ICOS) or negative (e.g. PD-L1, PD-L2, B7-H3, B7-H4) co-stimulatory molecules, BTNs so far only have been found to act through co-inhibition [34-37]. In addition, polymorphisms in the human gene encoding for BTNL2 have been linked to a growing number of inflammatory diseases, e.g. sarcoidosis, myositis and inflammatory bowel disease [38-40]. Moreover, human BTN2A1 has been shown to modulate immature dendritic cells (DC) by binding to Dendritic Cell-Specific Intercellular adhesion molecule-3-Grabbing Non-integrin (DC-SIGN) [41], and *Btnl1* has been recently found to regulate interactions with intraepithelial γδT lymphocytes in the murine small intestine by suppressing pro-inflammatory mediators of the NFκB pathway, such as IL-6, IL-15, CXCL1, and CCL4 [42]. In addition, many members of the B7-homolog (B7-H) family, such as PD-1 and CTLA-4, are expressed on tumour cells in various cancers, where they can be exploited by the cancerous cells to escape from immune destruction and impede B7 ongoing immune processes [43,44]. The presence of the deletion and the subsequent absence or reduced expression of the encoded proteins would allow a stronger response against tumour cells, implying that the *BTNL8_BTNL3-del* allele could act as a positive modulator of anti-tumour immunity. However, co-inhibitory molecules such as CTLA-4, PD-1 and BTLA have been shown to be crucial for the prevention of autoimmunity and polymorphism or deficiency of these molecules are associated with genetic susceptibility to autoimmune diseases in human and mice [45]. Follow-up studies on the BTNL proteins will shed more light on their function in autoimmune diseases and cancer.

*BTNL8* and *BTNL3* are primate-specific genes, while *BTNL9* has a clear ortholog in mice. Human *BTNL8* and *BTNL3* expression is primarily restricted to tissues of the digestive tract and at a lower level to spleen, thymus and lung. In addition *BTNL3* is expressed in neutrophils and *BTNL8* in eosinophils and at a reduced amount in neutrophils. In contrast, *BTNL9* is mainly expressed in B cells and lymphoid organs, e.g. thymus, spleen, bone marrow and lymph nodes [46,47]. Due to the high sequence homology and the similar expression-profile of BTNL8 and BTNL3 it is possible that the new BTNL8*3 fusion-protein compensates for the BTNL8 and BTNL3 wild-type proteins. However, more information is needed about the functions of BTNL8, BTNL3 and BTNL8*3 and the pathways they are involved in. Since almost all members of the BTN and BTNL families are highly expressed in the intestine [46], it has been postulated that these proteins act through a combined immunosuppressive effect, rather than a big impact of individual molecules [48]. Therefore, it would be interesting to check for the consequences of the CNV in diseases associated with polymorphisms in BTN and BNTL genes, e.g. BTNL2 in Crohn’s disease or ulcerative colitis. Nevertheless, a review of the published GWAS data in these two disorders did not reveal an association with the potential tagging SNPs for the deleted allele (rs2387715, rs4700772 or rs10063135), although these SNPs are present in the affy 6.0 (rs2387715) and Illumina Omni 1 and human 1 M arrays (rs4700772 and rs10063135. There is, nevertheless, a replicated association on Chron’s disease to the 5q35 region around *CPEB4* gene [49,50], about 3 Mb away from the deletion.

Interestingly, when investigating gene expression changes with regard to the *BTNL8_BTNL3-del* allele, we found TNF and the ERK1/AKT pathway to be central hubs of the network influenced by the deletion CNV in LCLs. TNF, ERK1 and AKT are important players in signal transduction pathways and key components of the immune response in humans and even a slight deregulation of those proteins might have an important impact in the response to pathogens. However, even though the HapMap repository represents a fantastic source for genetic studies, the analysis was limited due to the use of LCLs, which might not be the main cell-type where the BTNL8*3 CNV affects expression levels, since *BTNL3* and *BTNL8* are predominantly expressed in the digestive tract. Follow-up studies using other cell types will be needed.

## Conclusion

In summary, we provide a broad, functional analysis of a common deletion variant at several levels. We demonstrate the existence of a new fusion-protein, implying functional consequences of the CNV. Moreover, we proof structural changes at the immediate neighborhood of the CNV likely due to a position effect, as well as overall changes in the general expression-level of several genes involved in immune regulation and proliferation. Although the exact molecular function of BTNL8, BTNL3 and BTNL9 remains unknown, the high frequency of the deletion in some populations, its structural homology to B7 proteins and its tissue distribution make the CNV a potentially interesting candidate for diseases associated with infection and inflammation, especially in the gut.

## Methods

### Samples

1,103 samples from the International HapMap Phase III project, 1,007 individuals from the CEPH-HDGP cohort, and 2,000 Spanish samples were genotyped [51,52]. The samples consisted of 120 individuals from Utah with northern and western European ancestry (CEU); 90 Han Chinese from Bejing (CHB); 90 Japanese from Tokyo (JPT); 120 Yoruba form Ibadan, Nigeria (YRI); 43 individuals with African ancestry from Southwest USA (ASW); 100 Han Chinese from Denver, Colorado (CHD); 100 Gujarati Indians from Houston, Texas (GIH); 100 Luhya from Webuya, Kenya (LWK); 90 of Mexican ancestry from Los Angeles, California (MEX); 150 Maasai from Kinyawa, Kenya (MKK); 100 Toscans from Italy (TSI); and an independent control cohort of 2,000 Spanish unrelated individuals. The analysis from the CEPH-HGDP includes individuals from 51 different populations and excludes samples previously identified as duplicates as well as the genotypically abnormal samples 770 and 980 [53]. Individual genotypes are provided in Additional file 1: Table S1 and Table S2.

### Genotyping and sequence analysis

To identify possible CNVs, we used the Human Genome CGH Microarray Kit (Agilent 244 k aCGH microarray kit) with covers the genome with a 10-kb resolution between probes. aCGH was performed according to manufacturer’s protocol and as described previously [54]. Results obtained for the 5q35.3 region were confirmed by MLPA analysis and long range PCR analysis. Sequences of MLPA probes used in the study are listed in Additional file 1: Table S5.

### Statistical analysis

Linkage disequilibrium was calculated and visualized with Haploview. Statistical differences of allelic distributions among populations were assessed performing a chi-square test.

### PCR genotyping assay

Two allele-specific PCR assays using fluorescent oligonucleotides, were designed to distinguish non-deletion and deletion alleles based on primer sequences: non-del forward: 5′-(HEX)GGCACAACCCAGAACAAAGT-3′, non-del-reverse: 5′-TGAGAACCAAAATGAGCACAA-3′, del-forward: 5′-TTCCATGAACACCACCAAGA-3′ and del-reverse: 5′-(HEX)GACACAGGAGTGTGCAAGGT-3′. DNA from HapMap and CEPH-HGDP samples was available in the lab. Quality of DNA was checked on a 1% agarose gel prior PCR and samples with degraded DNA were excluded from the analysis. PCR was carried out in a GeneAmp PCR System (Applied Biosystems) using 100 ng of genomic DNA/reaction. Conditions consist of an initial denaturation step of 1 min at 95°C, 32 cycles of 30 s at 95°C denaturation, 30 s annealing at 62.5°C and 25 s elongation at 72°C and a final extension step at 72°C for 25 min. Non-deletion primers were located inside the deleted sequence and generated a 420-bp PCR product. Deletion primers amplified across the deletion breakpoints and resulted in a 340-bp product.

### Real-Time PCR analysis

Total mRNA was extracted from cells using the miRNA easy Kit (Qiagen), samples were treated with DNase I (Qiagen) for 15 min and 1 to 2 μg of RNA was reverse transcribed using the Superscript VILO kit (Invitrogen) according to the manufacturer’s protocol. Real time PCR was carried out using the Light cycler 480 from Roche. The PCR reaction contained 35 ng of cDNA, 10 pmol of each of the specific primers and 5 μl SYBR Green master mix in a final reaction volume of 10 μl. All reactions were performed in triplicates. Thermal-cycling conditions consisted of an initial denaturation of 10 min at 98°C, 40 cycles of 15 s at 95°C denaturation, 15 s annealing, and 18 s elongation at 72°C, and final extension at 72°C for 10 min. The optimal annealing temperature was determined for each oligonucleotide-pair individually. Cumulative fluorescence was measured after each of the 40 cycles. Product specific amplification was confirmed by melting curve analysis. Oligonucleotide sequences used for quantification are listed in Additional file 1: Table S6. Relative quantification of gene expression was determined by the construction of a relative expression calibration curve using serial dilutions of *ACTB* and *GAPDH* as a positive control.

### Allele-specific expression profiling

RNA extraction, DNase treatment, first-strand cDNA synthesis and qPCR were done as described above with *BTNL8* 5′-TTTGGCATTGTTGGACTGAA-3′ as forward primer and *BTNL3* 5′-ACACTCCCACATACCACCCT-3′ and *BTNL8* 5′-TCCTTCCTCCTGTCCACATC-3′ reverse primers.

### Western blot

Cell lysates were prepared as previously described [55]. Briefly, equal amounts of proteins (300–350 μg) were resolved by NuPAGE (4-12%; Invitrogen) and transferred to nitrocellulose membranes. Proteins were then blocked by incubation in 10% dry milk in TBST (0.1% Tween-20 in TBS) and probed with the indicated Antibody. Blots were then developed by enhanced chemiluminescence (ECL; Amersham).

### Antibodies

A polyclonal BTNL3 antibody was generated to peptides YIQHAMYDEEKGTPI, PPSTPPTRVGVFLDYE and YWVLRLTTEHLYFTF, corresponding to the cytoplasmic region of human BTNL3, according to standard procedures. Briefly, the three synthetic peptides were independently keyhole limpet hemocyanin (KLH)-conjugated, pooled, and injected into two female New Zealand White rabbits for antisera production. Pre-immune serum was collected before immunization and was used as a negative control. Antisera collected from the immunized rabbits were verified for reactivity by ELISA against the original peptides and the human BTNL3 protein. Rabbit BTNL9 antibody was purchased from Abcam (ab87049). Mouse Tubulin antibody was purchased from Santa Cruz (sc-5286).

### Expression analysis and Ingenuity Pathway Analysis software

Microarray expression data were obtained from Stranger et al., [15] and analyzed for differences in the expression level regarding the *BTNL8*/*BTNL3* genotype. The list of genes found to be influenced by the BTNL8*3 deletion CNV was submitted to Ingenuity Pathway Analysis (IPA; Ingenuity® Systems, Redwood City, CA, http://www.ingenuity.com) to identify common biological pathways. In addition, gene expression values were included in the input to identify up and down regulated genes in the pathways.

## Competing interests

The authors declare that they have no competing interests.

## Authors’ contributions

JA, EM and XE designed the study. JA and SV performed all experimental work. JA wrote the manuscript. JA and RR analyzed the data. RR performed the statistical analysis. JR and JJC collected data and participated in their interpretation. All authors read and approved the final manuscript.

## Supplementary Material

Additional file 1 Figure S1 Protein alignment of BTNL8 and BTNL3. BTNL8 and BTNL3 share 68.5% similarity in their amino-acid sequences. Segmental duplication, where cross-over occurred, is highlighted in yellow. **Table S1.** Individual genotyping HapMap. **Table S2.** Individual genotyping CEPH-HGDP. **Table S3.** Frequency of deletion by continental groups. **Table S4.** CEPH-HGDP genotype frequencies by geographic location. **Table S5.** Sequences of MLPA probes. **Table S6**. Sequences of oligonucleotides. Click here for file
