# Supplementary figures and images for: A common 56-kilobase deletion in a primate-specific segmental duplication creates a novel butyrophilin-like protein
Source: BMC Genet. 2013 Jul 6;14:61. doi: 10.1186/1471-2156-14-61 (PMC3729544; doi:10.1186/1471-2156-14-61)

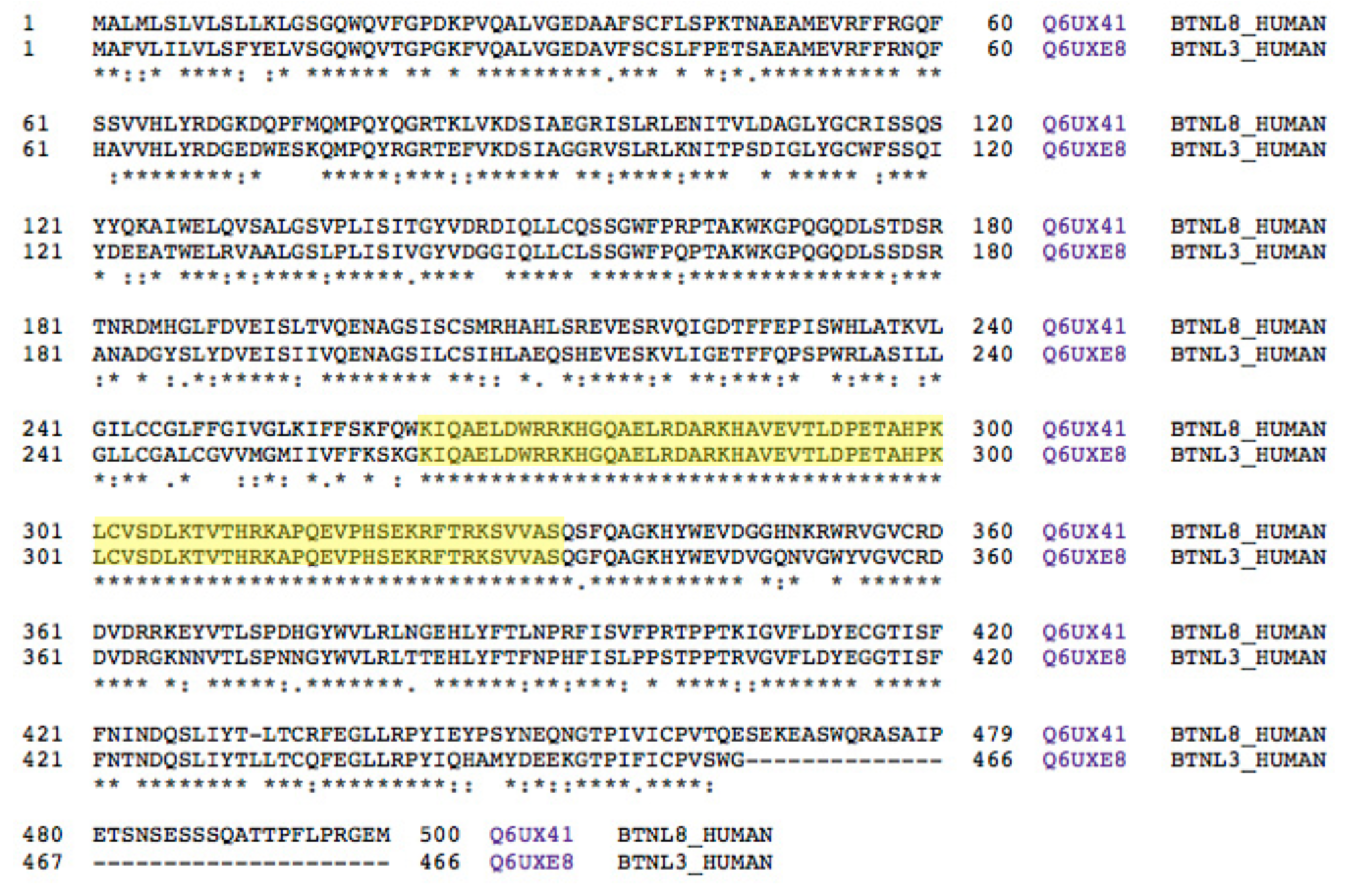

Supplement: Additional file 1 Figure S1 — Protein alignment of BTNL8 and BTNL3. BTNL8 and BTNL3 share 68.5% similarity in their amino-acid sequences. Segmental duplication, where cross-over occurred, is highlighted in yellow. Table S1. Individual genotyping HapMap. Table S2. Individual genotyping CEPH-HGDP. Table S3. Frequency of deletion by continental groups. Table S4. CEPH-HGDP genotype frequencies by geographic location. Table S5. Sequences of MLPA probes. Table S6. Sequences of oligonucleotides. [file 1471-2156-14-61-S1.zip › Supplement/Aigner_FigureS1.tif]
