# Supplementary material for: A common 56-kilobase deletion in a primate-specific segmental duplication creates a novel butyrophilin-like protein
Source: BMC Genet. 2013 Jul 6;14:61. doi: 10.1186/1471-2156-14-61 (PMC3729544; doi:10.1186/1471-2156-14-61)
Supplement: Additional file 1 Figure S1 — Protein alignment of BTNL8 and BTNL3. BTNL8 and BTNL3 share 68.5% similarity in their amino-acid sequences. Segmental duplication, where cross-over occurred, is highlighted in yellow. Table S1. Individual genotyping HapMap. Table S2. Individual genotyping CEPH-HGDP. Table S3. Frequency of deletion by continental groups. Table S4. CEPH-HGDP genotype frequencies by geographic location. Table S5. Sequences of MLPA probes. Table S6. Sequences of oligonucleotides. [file 1471-2156-14-61-S1.zip › Supplement/Aigner_Supplement.zip!/Aigner_TableS3.doc]

**Supplementary Table 3:** Frequency of *BTNL8/3* deletion by major continental groups

|  | Allele Counts (HGDP) | |  | Allele Counts (HapMap) | |  |
| --- | --- | --- | --- | --- | --- | --- |
| Geographic Region | Deletion | Insertion | Deletion Frequency | Deletion | Insertion | Deletion Frequency |
| Oceania | 2 | 76 | 0.03 | - | - | - |
| East Asia | 120 | 358 | 0.25 | 155 | 389 | 0.29 |
| Central Asia | 47 | 339 | 0.12 | - | - | - |
| Middle East | 60 | 184 | 0.25 | - | - | - |
| Europe | 121 | 191 | 0.39 | 145 | 271 | 0.35 |
| North Africa | 14 | 46 | 0.23 | - | - | - |
| Sub-Saharan Africa | 25 | 173 | 0.13 | 63 | 701 | 0.08 |
| America | 62 | 100 | 0.38 | 102 | 346 | 0.23 |
| **Total** | **451** | **1467** | **0.24** | **465** | **1707** | **0.22** |
