# Supplementary material for: A common 56-kilobase deletion in a primate-specific segmental duplication creates a novel butyrophilin-like protein
Source: BMC Genet. 2013 Jul 6;14:61. doi: 10.1186/1471-2156-14-61 (PMC3729544; doi:10.1186/1471-2156-14-61)
Supplement: Additional file 1 Figure S1 — Protein alignment of BTNL8 and BTNL3. BTNL8 and BTNL3 share 68.5% similarity in their amino-acid sequences. Segmental duplication, where cross-over occurred, is highlighted in yellow. Table S1. Individual genotyping HapMap. Table S2. Individual genotyping CEPH-HGDP. Table S3. Frequency of deletion by continental groups. Table S4. CEPH-HGDP genotype frequencies by geographic location. Table S5. Sequences of MLPA probes. Table S6. Sequences of oligonucleotides. [file 1471-2156-14-61-S1.zip › Supplement/Aigner_Supplement.zip!/Aigner_TableS4.doc]

**Supplementary Table 4:** HGDP Genotype Frequencies by Geographic Location

|  |  | **Genotype** | | |  |  |
| --- | --- | --- | --- | --- | --- | --- |
| **Population** | **Geographic Origin** | **D/D** | **D/I** | **I/I** | **Minor allele frequency** | **HWE^a^** |
| Biaka Pygmies | Central African Republic | 0 | 5 | 16 | 0.12 | 0.536 |
| Mbuti Pygmies | Democratic Republic of Congo | 0 | 1 | 5 | 0.08 | 0.824 |
| Mandenka | Senegal | 0 | 0 | 12 | 0 | - |
| Yoruba | Nigeria | 0 | 6 | 10 | 0.19 | 0.356 |
| Bantu N.E. | Kenya | 0 | 2 | 8 | 0.1 | 0.725 |
| San | Namibia | 0 | 1 | 6 | 0.7 | 0.839 |
| Bantu | South Africa | 0 | 10 | 17 | 0.19 | 0.238 |
| Mozabite | Algeria (Mzab) | 2 | 10 | 18 | 0.23 | 0.708 |
| Bedouin | Israel (Negev) | 0 | 10 | 17 | 0.19 | 0.238 |
| Druze | Israel (Carmel) | 2 | 17 | 26 | 0.23 | 0.708 |
| Palestinian | Israel (Central) | 4 | 21 | 25 | 0.29 | 0.888 |
| Brahui | Pakistan | 0 | 4 | 21 | 0.08 | 0.664 |
| Balochi | Pakistan | 0 | 3 | 22 | 0.06 | 0.750 |
| Hazara | Pakistan | 0 | 7 | 15 | 0.08 | 0.375 |
| Makrani | Pakistan | 0 | 10 | 15 | 0.2 | 0.2113 |
| Sindhi | Pakistan | 0 | 4 | 20 | 0.08 | 0.656 |
| Pathan | Pakistan | 0 | 11 | 14 | 0.22 | 0.158 |
| Kalash | Pakistan | 1 | 5 | 19 | 0.14 | 0.397 |
| Burusho | Pakistan | 0 | 1 | 21 | 0.02 | 0.913 |
| Han | China | 1 | 19 | 25 | 0.23 | 0.227 |
| Tujia | China | 0 | 2 | 6 | 0.13 | 0.686 |
| Yizu | China | 1 | 3 | 6 | 0.25 | 0.527 |
| Miaozu | China | 0 | 2 | 8 | 0.1 | 0.725 |
| Oroqen | China | 0 | 3 | 6 | 0.17 | 0.549 |
| Daur | China | 1 | 2 | 7 | 0.2 | 0.235 |
| Mongola | China | 3 | 2 | 5 | 0.35 | 0.281 |
| Hezhen | China | 0 | 10 | 0 | 0.5 | 0.002 |
| Xibo | China | 1 | 1 | 8 | 0.15 | 0.055 |
| Uygur | China | 0 | 2 | 8 | 0.1 | 0.725 |
| Dai | China | 0 | 2 | 3 | 0.2 | 0.576 |
| Lahu | China | 0 | 3 | 7 | 0.15 | 0.577 |
| She | China | 0 | 1 | 6 | 0.07 | 0.839 |
| Naxi | China | 0 | 1 | 7 | 0.06 | 0.850 |
| Tu | China | 0 | 5 | 5 | 0.25 | 0.292 |
| Yakut | Siberia | 5 | 11 | 9 | 0.42 | 0.628 |
| Japanese | Japan | 6 | 13 | 12 | 0.4 | 0.474 |
| Cambodian | Cambodia | 1 | 0 | 10 | 0.09 | 0.001 |
| Papuan | New Guinea | 0 | 0 | 17 | 0 | - |
| NAN Melanesian | Bougainville | 0 | 2 | 20 | 0.15 | 0.823 |
| French | France | 3 | 15 | 11 | 0.36 | 0.519 |
| French Basque | France | 5 | 15 | 4 | 0.52 | 0.217 |
| Sardinian | Italy | 5 | 14 | 9 | 0.43 | 0.912 |
| North Italian | Italy (Bergamo) | 3 | 2 | 9 | 0.29 | 0.015 |
| Tuscan | Italy | 0 | 4 | 4 | 0.25 | 0.346 |
| Orcadian | Orkney Islands | 1 | 12 | 3 | 0.44 | 0.036 |
| Adygei | Russia Caucasus | 1 | 3 | 12 | 0.26 | 0.249 |
| Russian | Russia | 8 | 4 | 9 | 0.48 | 0.461 |
| Pima | Mexico | 2 | 11 | 4 | 0.44 | 0.198 |
| Maya | Mexico | 1 | 11 | 8 | 0.33 | 0.257 |
| Colombian | Colombia | 2 | 4 | 3 | 0.44 | 0.764 |
| Karitiana | Brazil | 4 | 7 | 4 | 0.5 | 0.796 |
| Surui | Brazil | 2 | 7 | 11 | 0.28 | 0.585 |

Numbers of individuals genotyped are given:

(D/D) homozygous for deletion

(I/D) hemizygous for insertion

(I/I) homozygous for insertion

^a^Probability of Hardy-Weinberg equilibrium
